# Capillary versus arterial blood gases. Accuracy, acceptability, and suitability for use during sleep studies to calibrate transcutaneous carbon dioxide measurement

**DOI:** 10.1007/s11325-026-03739-3

**Published:** 2026-07-08

**Authors:** Nicole L. Sheers, Linda Rautela, Steven James Lindstrom, Krisha Saravanan, Jennifer Cori, Danny Brazzale, Elisa San Pedro, Michelle Mason, Peter D. Rochford, Christine F. McDonald, David J. Berlowitz, Mark E. Howard

**Affiliations:** 1 Institute for Breathing and Sleep, Melbourne, Australia; 2 Department of Physiotherapy, University of Melbourne, Melbourne, Australia; 3 Department of Physiotherapy, Austin Health, Melbourne, Australia; 4 Deakin University, Geelong, VIC Australia; 5 Departments of Intensive Care; Respiratory and Sleep Medicine, Mercy Health, Melbourne, Australia; 6 Department of Respiratory and Medicine, Alfred Health, Melbourne, Australia; 7 Department of Intensive Care Medicine, Epworth Healthcare, Melbourne, Australia; 8 Barwon Health, Geelong, VIC Australia; 9 Department of Respiratory and Sleep Medicine, Austin Health, Melbourne, Australia; 10 Melbourne School of Psychological Sciences, University of Melbourne, Parkville, VIC Australia; 11 Department of Medicine, University of Melbourne, Melbourne, Australia

**Keywords:** Transcutaneous carbon dioxide, Capillary blood gas, Arterial blood gas, Polysomnography

## Abstract

**Purpose:**

To determine whether capillary blood gas carbon dioxide values can substitute for arterial sampling to calibrate transcutaneous carbon dioxide during polysomnography.

**Methods:**

Participants underwent capillary (arterialized earlobe) and blood gas (radial artery) sampling at polysomnography commencement (Evening) and end (Morning). Participant and scientist sample preference, and participant pain were recorded. The transcutaneous offset from the corresponding arterial/capillary sample, and the transcutaneous electrode drift over the night were determined.

**Results:**

Seventy-five people consented and 44 had complete paired timepoint data. Both the capillary and arterial carbon dioxide values were higher than the transcutaneous (capillary evening mean -0.9 ± standard deviation 2.8, capillary morning -2.5 ± 3.9, arterial evening -3.7 ± 2.7, arterial morning -5.6 ± 3.8 mmHg). Capillary and arterial measures were comparable for assessing transcutaneous carbon dioxide drift (mean difference -0.3 mmHg (95% confidence interval -1.3, 0.7). The failure rate for both techniques was equivalent. Patients and scientists both preferred the capillary method.

**Conclusion:**

Capillary blood gas sampling of carbon dioxide levels from the earlobe is feasible. Capillary sampling provides a comparable measure of transcutaneous carbon dioxide drift, but not absolute offset, during polysomnography to that observed with radial arterial sampling. Importantly, patients prefer the capillary technique as it is less painful.

**Supplementary Information:**

The online version contains supplementary material available at 10.1007/s11325-026-03739-3.

## Introduction

Alveolar ventilation changes as we pass between wake, non- and rapid eye movement sleep stages. Understanding ventilation during sleep requires quantification of these sleep-stage changes in oxygen and carbon dioxide levels. An arterial blood gas sample (ABG) is the definitive measure of ventilation, but an ABG is a snapshot, not a continuous measure and can be both painful and technically difficult [1–3]. Alternative tests, such as venous blood gases (VBG) or arterialised capillary blood gases (CAPgas), while still spot measures, are purported to be easier to perform and less uncomfortable for the patient [4, 5]. 

A recent systematic review examined ABG versus VBG measures across a range of participant groups [6]. Of the 22 included studies, less than a quarter found a strong correlation between ABG and VBG, and there was substantial disagreement regarding the precision, appropriateness, and utility of VBG as an alternative to ABG. It is thus unclear whether a VBG can substitute for an ABG, and in what clinical situations.

The CAPgas is typically obtained from an earlobe puncture rather than direct arterial or venous sampling. A CAPgas may be easier to perform and more comfortable for the patient, particularly when an ABG is technically difficult [7]. Previous comparisons have suggested that CAPgas might substitute for ABGs for measurement of pH and arterial carbon dioxide (PaCO_2_), although not for oxygen (PaO_2_) due to very wide limits of agreement [7]. These data are however very heterogeneous, and the majority of these studies were performed for oxygen therapy assessment, on acutely unwell patients in adult emergency or intensive care, or in paediatrics [5, 8]. 

Laboratory polysomnography (PSG) provides comprehensive testing of sleep disordered breathing, but unfortunately the spot nature of ABG, VBG, and CAPgas fundamentally limits their utility to continuously measure ventilation efficacy during PSG. Transcutaneous carbon dioxide (TcCO_2_) monitoring is a continuous, non-invasive method of monitoring changes in ventilation. TcCO_2_ monitoring during sleep is recommended by the American Association of Sleep Medicine (AASM) for assisting with the diagnosis of sleep-related hypoventilation [2, 9]. While continuous TcCO_2_ monitoring has clear advantages over spot blood gas CO_2_ measurements, absolute TcCO_2_ values at any moment in time may be variably offset from the PaCO_2_ [1, 2, 10]. 

Despite the potential utility of TcCO_2_ for measuring alveolar ventilation, there may be an offset from PaCO_2_ at application and a change in offset over time, with TcCO_2_ drift defined as any change in the offset across the monitoring period. Our group has previously demonstrated that an ABG measure of CO_2_ at the start and the end of extended monitoring can correct for both offset and drift, such that the TcCO_2_ tracks PaCO_2_ with minimal residual bias and further, that drift is linear [9]. TcCO_2_ can thus reflect alveolar CO_2_ as it changes across sleep, but only if the TcCO_2_ is anchored with a CO_2_ measure at either end of the study to enable correction for bias in the evening and morning. The utility of the CAPgas as an alternative TcCO_2_ reference value to an arterial CO_2_ has not been previously reported, and therefore the primary aim of this study was to determine whether CAPgas-derived pCO_2_ values can substitute for ABG-derived pCO_2_ to calibrate and correct the TcCO_2_ during PSG. Secondary aims were:

- to examine the agreement between ABG and CAPgas parameters of pH, pCO_2_ and pO_2_ in a stable adult population with suspected or confirmed chronic ventilatory failure,
- to determine if CAPgas samples can be obtained more consistently than ABG samples and if the time to obtain a successful CAPgas or ABG is different,
- to compare participant perception of pain with both ABG and CAPgas sampling using a self-rating scale, and.
- to compare operator and participant preference between ABG and CAPgas sampling.

## Methods

### Study design

A prospective, single-centre, comparison of ABG versus CAPgas, and their relationships to TcCO_2_ was conducted. Ethical approval was obtained from the institution’s research ethics committee (HREC/16/Austin/441). The trial was prospectively registered at anzctr.org.au (ACTRN12617000562370) [11]. 

Potential adult participants undergoing clinically indicated PSG with planned TcCO_2_ monitoring at the Austin Hospital were recruited sequentially. Exclusion criteria included pregnancy, inability to understand English or unavailability of CAPgas-trained staff on the study night. All participants provided written informed consent (or verbal plus witness-signed consent if unable to sign).

### Procedure

Participants underwent ABG and CAPgas sampling at PSG commencement (Evening) and completion (Morning). Sampling type order was randomized using a computer-generated sequence (www.randomization.com) and applied to both timepoints (Order 1: CAPgas then ABG; Order 2: ABG then CAPgas). Allocation concealment was ensured using sealed opaque envelopes.

At least 10 min prior to blood sampling, the topical anaesthetic and vasodilatory cream Finalgon^®^ (butoxyethyl nicotinate 10.8 mg and nonivamide 1.7 mg) was applied. For CAPgas sampling the participant-preferred earlobe was punctured using a lancet (BD Microtainer Blue, Becton Dickinson Pty Ltd) and the sample collected in a heparinised plastic capillary tube (75–95 µl). For ABG sampling, the radial artery was punctured and the sample collected using a 3 ml heparinised syringe. Sleep laboratory scientists were skilled at ABG sampling and underwent training in CAPgas sampling. Scientists were instructed to take the shortest possible time between sampling procedures. Samples were analysed in the same order they were collected using an ABL90FP blood gas analyser (Radiometer, Copenhagen, Denmark) on site, negating the need for sample cooling.

Participant pain ratings for each procedure were collected using an 11-point Likert scale. Participant and scientist test preference (ABG or CAPgas) were recorded for each timepoint. Notes were made if samples were unable to be collected or technical issues prevented sample analysis.

TcCO_2_ was monitored using a Radiometer TCM4-CombiM device, with a “tc Sensor 54’ electrode placed below the clavicle, the sensor temperature set to 42 °C and calibration performed as per manufacturer’s instructions (Radiometer Medical ApS, Denmark). The TcCO_2_ value when each sample was obtained was recorded (TcCO_2 ABG_ and TcCO_2 CAP_). The offset was defined as the difference between the TcCO_2_ and the corresponding arterial/capillary sample (ABG Offset = TcCO_2 ABG_ minus PaCO_2_; CAPgas Offset = TcCO_2 CAP_ minus P_CAP_CO_2_). The drift was defined as the Morning Offset (end of PSG) minus the Evening Offset for each pair of TcCO_2_ and CAPgas or ABG values (ABG Drift = ABG Offset Morning minus Evening; CAPgas Drift = CAPgas Offset Morning minus Evening).

#### Statistical analyses

Demographic data are summarised as mean ± standard deviation or frequency (count), as appropriate. The ABG and CAPgas samples were compared using paired t-tests, with results presented as mean difference (95% confidence interval, CI). Agreement was illustrated with Bland Altman plots and assessed using Intraclass Correlation Coefficients (two-way mixed effects model: ICC (3,1)). Parametric inferential statistics were performed on participant data with paired ABG and CAPgas samples at both evening and morning timepoints. Non-paired data were likely not “missing at random”, and thus biases could have been introduced with imputation.

T-tests and Bland Altman plots assessed the difference in offset between the two sampling methods (Evening ABG Offset and CAPgas Offset) and the difference in drift (ABG Drift minus CAPgas Drift) over the monitoring period.

Feasibility of the different techniques was assessed via:

- number of successful samples (both obtained and analysed).
- number of attempts required to obtain an adequate sample.
- time taken to obtain an adequate sample (sampling duration).
- pain as reported on an 11-point Likert scale (0 = no pain, 10 = maximal pain).
- staff experience, quantified by the sample sequence number (higher = sample obtained later in the experiment’s course).
- staff preference for each sampling technique in each participant.
- participant preference for each sampling technique.

Chi-square tests for homogeneity assessed if the number of unsuccessful samples, and the number of attempts needed to obtain a successful sample, were different between methods. Linear mixed models, including participant as a fixed variable, assessed the effect of sampling method (ABG or CAPgas), timepoint (Evening or Morning), and sampling order on sampling duration and pain. Binomial testing assessed participant and scientist preference for a sampling method, with a null hypothesis of equal (50%) technique preference. Logistic regression modelling assessed the influence of number of attempts, sampling duration, pain and scientist experience on participant and scientist preference for a particular sampling method.

Statistical analyses were performed using Stata^®^/IC 15.1 for Mac (StataCorp LLC, Texas, USA).

## Results

**Fig. 1:**
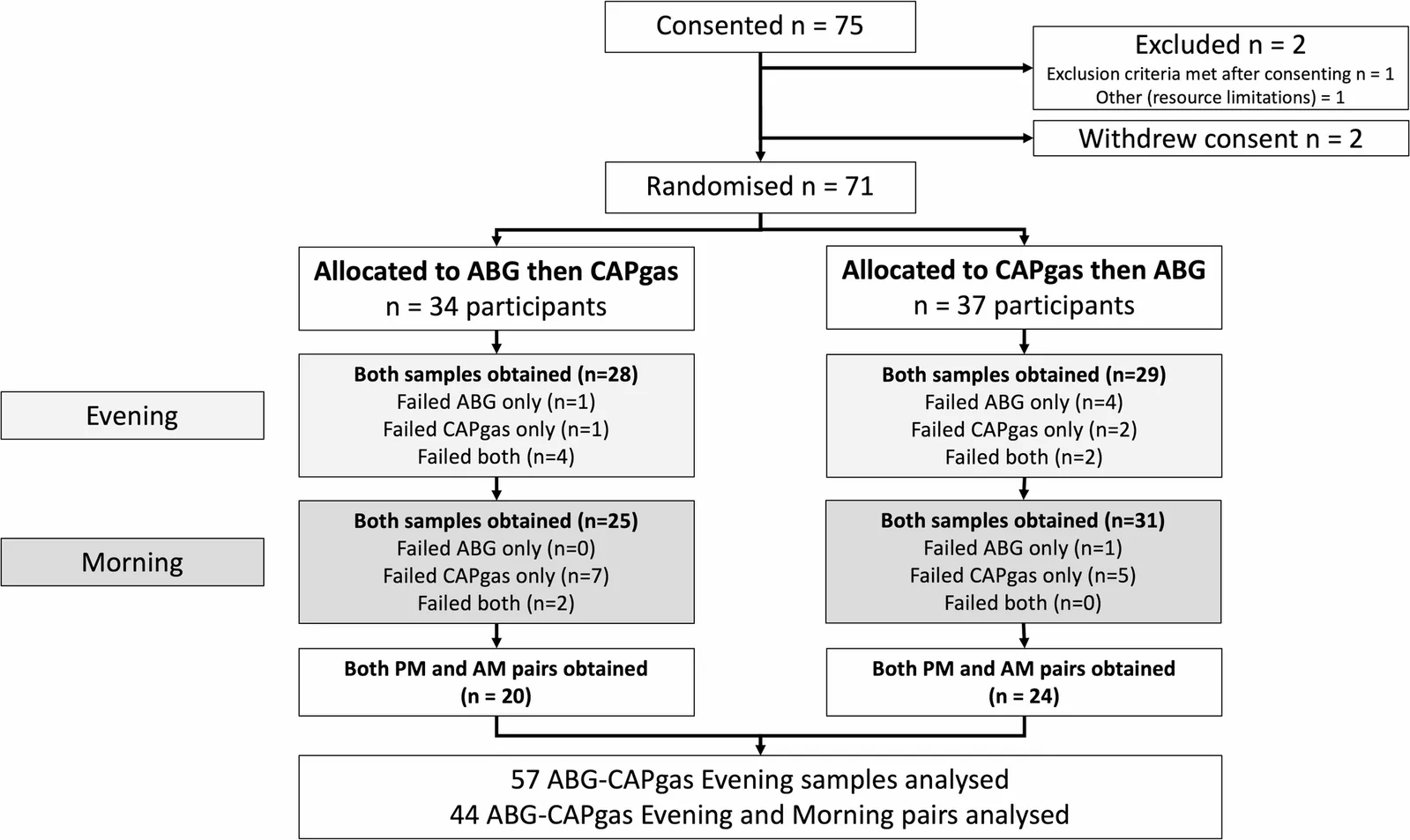
Study participant flow. ABG = arterial blood gas; CAPgas = arterialised capillary blood gas; PM = Evening; AM = Morning

Between 17th September 2018 and 20th April 2019, 75 participants were recruited (Fig. 1). Of the 71 randomised participants (Table 1), 44 had paired CAPgas and ABG data obtained at both Evening and Morning timepoints (20 with Order 1).

**Table 1:** Participant demographics, diagnosis, and polysomnography (PSG) type

Variable	*N* = 71 all randomised participants	*N* = 44 complete, paired data
Age (years)	58.5 ± 16.3	59.4 ± 15.6
Sex (M : F)	40 : 31	24 : 20
BMI (kg/m^2^) [n]	34.9 ± 17.5 [68]	36.2 ± 15.3 [43]
Diagnosis		
OSA/OHS	12 (17%)	9 (20%)
MND/ALS	18 (25%)	9 (20%)
Slow NMD	18 (25%)	8 (18%)
Obstructive lung disease (COPD)	13 (18%)	12 (27%)
“Other” (Restrictive chest wall disease/central hypoventilation)	10 (14%)	6 (14%)
Type of PSG		
Diagnostic	10 (14%)	3 (7%)
CPAP	7 (10%)	7 (16%)
NIV	49 (69%)	30 (68%)
Split CPAP/NIV	5 (7%)	4 (9%)
PaCO_2_ Evening (mmHg) [n]	48.0 ± 7.5 [60]	48.0 ± 7.3 [44]
PaCO_2_ Morning (mmHg) [n]	47.0 ± 7.8 [68]	47.4 ± 8.2 [44]

Data are presented as mean ± standard deviation or frequency count (percent of total cohort). *BMI* body mass index, *OSA/OHS* obstructive sleep apnoea/obesity hypoventilation syndrome, *MND/ALS* Motor Neurone Disease/Amyotrophic Lateral Sclerosis, *Slow NMD* Slowly progressive neuromuscular disease (includes: minicore myopathy, spinal muscular atrophy, acid maltase deficiency, Charcot Marie Tooth disease, Guillian-Barre syndrome); *COPD* chronic obstructive pulmonary disease; *CPAP* continuous positive airway pressure, *NIV* non-invasive ventilation. *PaCO*_2_ carbon dioxide on arterial blood gas.

Complete paired data refers to number of participants with ABG and CAPgas samples successfully obtained at both the Evening and Morning time points.

**Fig. 2:**
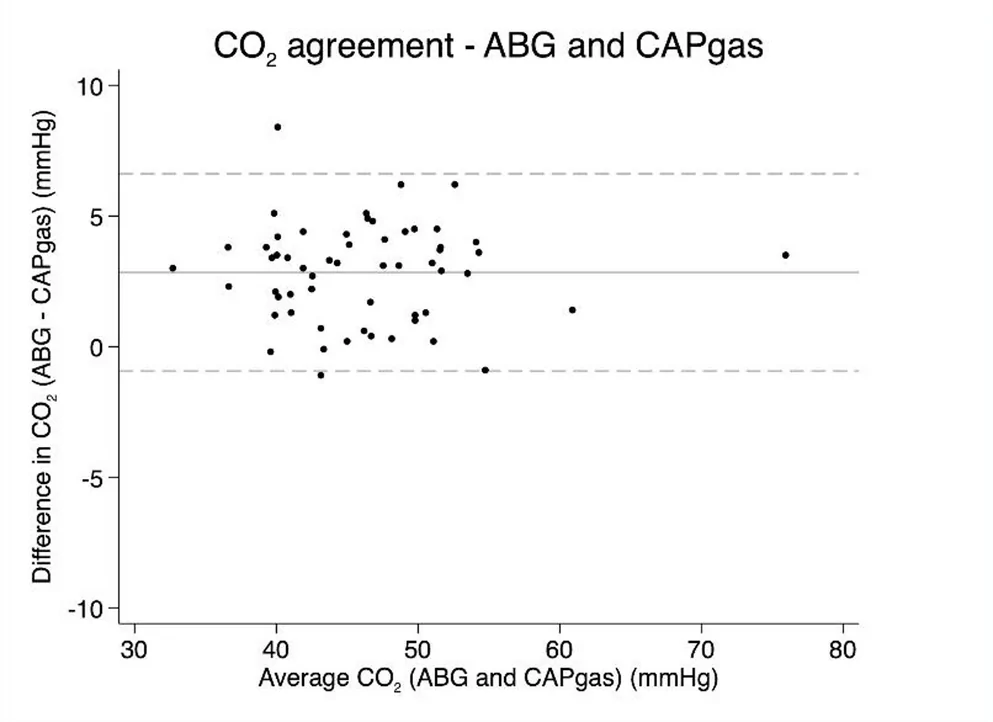
Bland Altman plots illustrating mean difference and limits of agreement between ABG and CAPgas blood parameters for CO_2_, based on paired samples obtained at the Evening session (*n* = 57). Mean bias (limits of agreement) = 2.83 (−0.87 to 6.53). Solid line represents mean bias (mean difference), dashed lines represent upper and lower levels of agreement (mean difference ± 2 standard deviations)

For the 57 participants with paired CAPgas and ABG Evening samples, the mean differences (95% CI) between arterial and capillary blood gas parameters were: pH −0.019 (−0.023, −0.016), *p* < 0.001; PCO_2_ = 2.8 (2.3, 3.3) mmHg, *p* < 0.001 and PO_2_ = −2.9 (−4.6, −1.1) mmHg, *p* = 0.002. There was a significant correlation between the two sampling methods for pH, CO_2_ and O_2_ values (ICC [95% CI] for pH = 0.90 [0.84, 0.94], *p* < 0.001; CO_2_ = 0.96 [0.94, 0.98], *p* < 0.001; O_2_ = 0.91 [0.85, 0.94], *p* < 0.001). Figure 2 illustrates PCO_2_ agreement between ABG and CAPgas. (Online Figure S1a & S1b for O_2_ and pH).

**Fig. 3:**
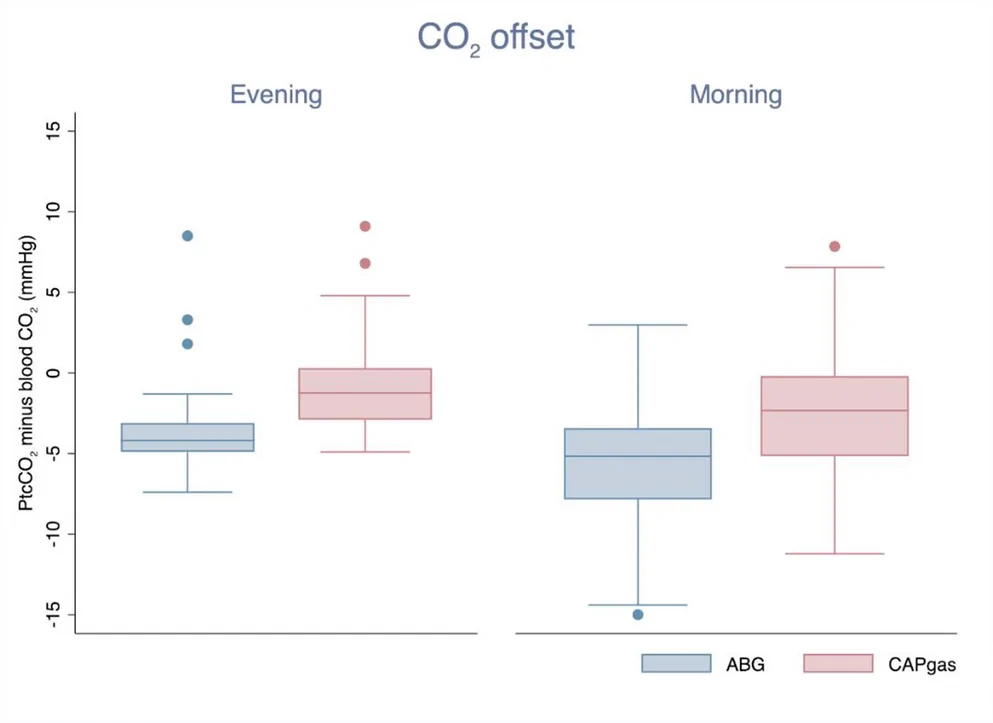
Comparison between ABG Offset and CAPgas Offset at Evening and Morning timepoints (*n* = 44). Box and whisker plots representing group median, quartiles, upper and lower adjacent values (1.5IQR) and outliers for CO_2_ Offset, the difference between transcutaneous and blood sampled carbon dioxide ABG Offset: PtcCO_2 ABG_ - PaCO_2_; CAPgas Offset: PtcCO_2 CAP_ - P_CAP_CO_2_). Data calculated from the n = 44 participants with paired arterial blood gas (ABG) and capillary blood gas (CAPgas) derived CO_2_ measurements at both sampling timepoints (Evening and Morning)

**Fig. 4:**
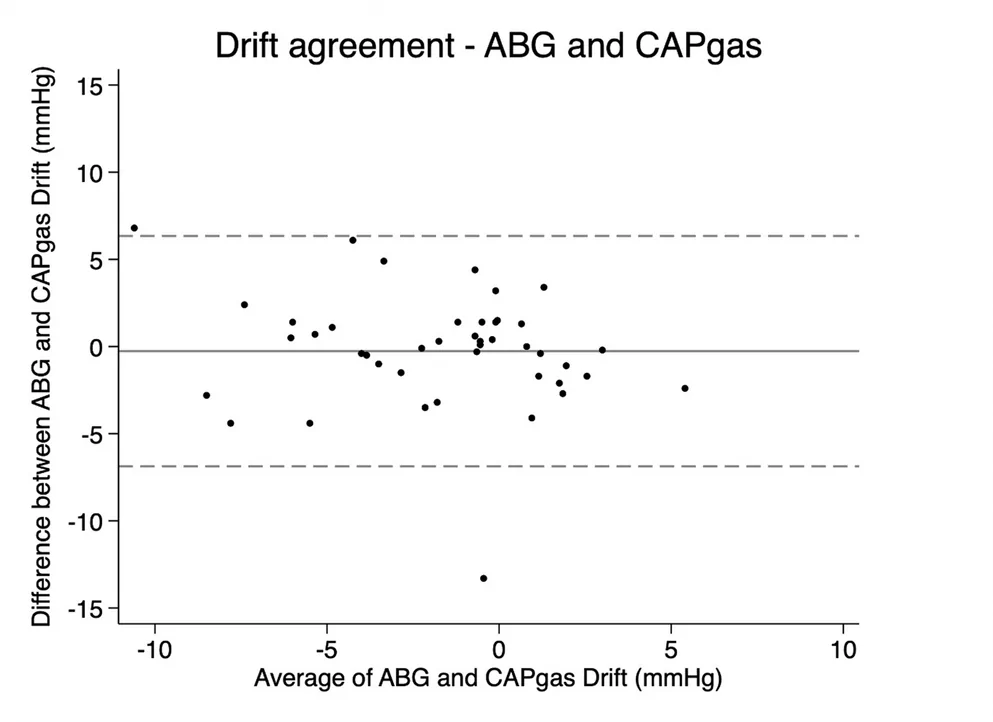
Bland Altman plot illustrating mean difference and limits of agreement between ABG Drift (ABG Offset Morning minus Evening samples) and CAPgas Drift (CAPgas Offset Morning minus Evening samples). Data are individual participant paired samples (*n* = 44). Solid line represents mean bias (mean difference: ABG Drift minus CAPgas Drift), dashed lines represent upper and lower levels of agreement (mean difference ± 2 standard deviations = −0.27 ± 6.60 [−6.87, 6.34])

Offset and Drift were calculated for the 44 participants with all ABG (PaCO_2_), CAPgas (P_CAP_CO_2_) and TcCO_2_ measures at both Evening and Morning (Table 2). The average absolute PaCO_2_ and P_CAP_CO_2_ values did not change over the night but the TcCO_2_ significantly decreased, resulting in larger absolute differences. There was a larger, negative Offset between TcCO_2_ and ABG compared to TcCO_2_ and CAPgas at both Evening and Morning (Table 2; Fig. 3, Online S2a, S2b and S3). The TcCO_2_ measure drifted further negatively from both the ABG and the CAPgas PCO_2_ values over the night, but there was no difference in the magnitude of these drifts between the sampling methods (Table 2; Fig. 4).

**Table 2:** Carbon dioxide values (mmHg) for the 44 participants with paired ABG and CAPgas samples at Evening and Morning timepoints (*n* = 44)

CO_2_ variable		Mean ± SD	Mean (95% CI)	*p*
PaCO_2_	Evening	48.0 ± 7.3		
	Morning	47.4 ± 8.2		
(Morning - Evening)	Change		−0.7 (−2.3, 0.9)	0.397
TcCO_2 ABG_	Evening	44.3 ± 8.3		
	Morning	41.8 ± 7.8		
(Morning - Evening)	Change		−2.6 (−3.9, −1.2)	< 0.001
ABG Offset (TcCO_2 ABG_ – PaCO_2_)	Evening	−3.7 ± 2.7		< 0.001
	Morning	−5.6 ± 3.8		< 0.001
P_CAP_CO_2_	Evening	45.2 ± 7.4		
	Morning	44.5 ± 7.9		
(Morning - Evening)	Change		−0.7 (−2.2, 0.9)	0.385
TcCO_2 CAP_	Evening	44.3 ± 8.2		
	Morning	42.0 ± 7.7		
(Morning - Evening)	Change		−2.3 (−3.6, −1.0)	0.001
CAPgas Offset (TcCO_2 CAP_ – P_CAP_CO_2_)	Evening	−0.9 ± 2.8		0.042
	Morning	−2.5 ± 3.9		0.001
Difference in Offsets (ABG – CAPgas)	Evening		−2.8 (−3.4, −2.2)	< 0.001
	Morning		−3.1 (−4.1, −2.1)	< 0.001
TcCO_2_ Drift based on ABG (ABG offset Morning – Evening)			−1.9 (−2.9, −0.8)	< 0.001
TcCO_2_ Drift based on CAPgas (CAPgas offset Morning – Evening)			−1.6 (−2.8, −0.4)	0.011
Difference in Drift (ABG – CAPgas)			−0.3 (−1.3, 0.7)	0.596

Data are presented as mean ± standard deviation and mean difference (95% confidence intervals). *PaCO*_2_ carbon dioxide value from the arterial blood gas sample (ABG), *PtcCO*_2 ABG_ transcutaneous carbon dioxide value at the time of ABG sampling, *P*_CAP_*CO*_2_ carbon dioxide value from capillary blood gas (CAPgas), *TcCO*_2 CAP_ transcutaneous carbon dioxide value at the time of CAPgas sampling.

### Feasibility

A total of 128 ABG and 119 CAPgas samples were successfully obtained (Fig. 1) from 71 randomised participants. Unsuccessful sampling reasons included: collection (CAPgas = 10, ABG = 9) and machine analysis errors (CAPgas = 7); participant declined (CAPgas = 4, ABG = 3) and protocol breaches (CAPgas = 2, ABG = 2). There was no difference in the number of unsuccessful occasions between blood sampling methods (c2 = 2.52, *p* = 0.113), timepoint (c2 = 0.28, *p* = 0.597) or order (c2 = 1.34, *p* = 0.247).

No difference was observed in the number of attempts required to obtain a successful sample. Sampling was successful on the first attempt in 99/128 (ABG) and 101/119 (CAPgas) occasions (χ2 = 4.16, *p* = 0.244). There was no correlation between the number of attempts and scientist experience for CAPgas (*r* = −0.08, *p* = 0.355), and a weak correlation for ABG (*r* = 0.39, *p* < 0.001).

### Characteristics of all successful and unsuccessful samples

The mean ± SD sampling duration for the ABG method was 138 ± 65 s when successful. ABG pain score was 3.7 ± 2.6 if successful and 7.3 ± 2.0 if unsuccessful (*n* = 7, missing data *n* = 7). For the CAPgas method, sampling duration was 101 ± 58 s when successful and 195 ± 84 s when unsuccessful (*n* = 10, missing data *n* = 13). CAPgas pain score was 2.0 ± 2.0 if successful and 3.1 ± 2.3 if unsuccessful (*n* = 15, missing data *n* = 8).

### Participant and scientist preferences

Comparisons between ABG and CAPgas methods were possible when both sampling methods were successful at either timepoint (*n* = 113 participants; Evening = 57, Morning = 56; Fig. 1).

A significant effect of sampling method on duration (p = < 0.001) was observed, with ABG taking a mean [95% CI] of 32 [18, 46] seconds longer than CAPgas (ABG = 136 [121, 152]; CAPgas = 104 [89, 120]). No effects or interactions with timepoint and sampling order were observed. There was a significant effect of sampling method on pain (p = < 0.001) with ABG rated more painful (1.4 [0.9, 1.9] points) than CAPgas; ABG 3.5 [2.9, 4.0]; CAPgas 2.1 [1.5, 2.6]). No effect or interaction between pain, timepoint and/or sampling order was observed.

Participants (84 occasions (75%)) and scientists (80 occasions (71%)) preferred the CAPgas to the ABG sampling (both *p* < 0.001). For the scientists, the odds of preferring CAPgas significantly increased as they became more practiced with CAPgas sampling (OR 1.07 [1.02, 113]; *p* = 0.012). CAPgas sampling duration and participant reported pain were included in the final overall regression model but they were not independently significant (sample duration OR 0.99 [0.98, 1.00], pain OR 0.81 [0.62, 1.07] Online Table S1). In contrast, participant preference was driven by pain experience alone. Experiencing pain on ABG increased the odds of preferring CAPgas by 2.22 [1.43, 3.48], *p* < 0.001 and similarly, experiencing pain during a CAPgas decreased the odds of preferring CAPgas by 0.52 [0.36, 0.76], *p* = 0.001.

## Discussion

This experiment analysed the utility of an earlobe arterialised capillary blood gas (CAPgas) to calibrate TcCO_2_ monitoring in a clinical sample of patients undergoing overnight PSG. We observed that CAPgas was comparable with an ABG for assessing drift in TcCO_2_, albeit slightly underestimating the absolute offset of TcCO_2_ in comparison to ABGs. The PCO_2_ value obtained via both capillary and arterial blood gas was higher than the TcCO_2_ value but lower for the P_CAP_CO_2_ to TcCO_2_ value than PaCO_2_ at both Evening and Morning timepoints. Both blood sampling techniques demonstrated drift in TcCO_2_ over the night with close limits of agreement that were within the 10mmHg considered to be clinically significant [2]. The average magnitude of this drift was small and similar between techniques, supporting the conclusion that CAPgas can substitute for ABG-derived CO_2_ to calibrate and correct TcCO_2_ drift, but not for assessing absolute TcCO_2_ offset during PSG. The failure rate for both techniques was equivalent, and both the patients and scientists preferred the capillary method.

### Feasibility and technique preferences

Arterialised capillary blood sampling is recognised as an alternative for direct arterial sampling. It can be safely performed without needing to locate an artery and is reportedly more acceptable to patients [12]. The earlobe sampling site has previously been shown to have closer agreement to arterial [8]. CAPgas and ABG samples were successfully obtained 84 and 90% of the time respectively in the current trial, suggesting both techniques are clinically feasible. Similar numbers of attempts were required for both, and neither technique was influenced by whether it was an evening or morning sample. Staff were highly experienced in the collection of ABGs and the learning effect of CApgas sampling expertise was demonstrated with sample collection time and number of attempts before a successful CAPgas decreasing over the study. Similarly, staff preference for CAPgas evolved as experience, familiarity and collection time improved.

On average, CAPgas sampling was less painful and over 30 s quicker than an ABG. An unsuccessful ABG attempt was the most painful, and even successful ABG samples were on average rated as more painful than both successful and unsuccessful CAPgas attempts. While both scientists and patients preferred the CAPgas as a technique, their reasons were different. Reported pain drove the patients’ preference for CAPgas, whereas increasing experience with the CAPgas technique underpinned the scientists’ preference.

This was a prospective single-centre study in those requiring PSG within a large and specialized home ventilation service. Despite this, only 44 of the 71 participants had paired samples for all their measures at both timepoints. There was little consistent difference between CAPgas and ABG failure rates or reasons, but a selection bias in our analysed data cannot be excluded. Similarly, other centres with differently experienced staff or patient populations may not demonstrate the same feasibility and acceptability outcomes as we observed.

### Accuracy and agreement between techniques: Offset

The “spot” or instantaneous TcCO_2_ was a mean of 3.7 ± 2.7 mmHg lower than the PaCO_2_, and 0.9 ± 2.8 lower than the P_CAP_CO_2_ at the start of monitoring, within the clinically significant difference of 10mmHg [2] and consistent with those from Storre et al. [13] and Aarrestad et al. [14] (Table 2; Fig. 4). Our limits of agreement spanned a similar magnitude (evening ABG offset 11 mmHg, evening CAPgas offset 11.4 mmHg) to those reported more recently by Aarrestad (8.3 mmHg) [14], and Storre (11.4 mmHg and 13.6) [15], but were smaller than earlier reports from Storre (17.1 mmHg) [13] and Stieglitz (26 mmHg) [16]. Our data support previous assertions that newer TcCO_2_ monitoring devices show improved average absolute agreement between PtcCO_2_ and PCO_2_, but wide variation persists, likely variably attributable to different devices, sensor placement and temperature, running time and individual participant characteristics such as subcutaneous fat.

### Accuracy and agreement between techniques: Drift

The TcCO_2_ drifted down from the reference values by an average of 1.6 (95% CI = 0.4, 2.8) mmHg (CAPgas) and 1.9 (0.8, 2.9) mmHg (ABG) over the course of the night (Table 2). This narrow range of drift is unlikely to change the overall interpretation of continuous CO_2_ monitoring during PSG, however as illustrated in Online Figures S4a and S4b, individual participants’ offset and thus drift values vary substantially. Aarrestad and colleagues found a smaller but comparable average drift (1.1 (95% CI = 0.1, 2.0) mmHg) using a similar device to ours from the same manufacturer [14]. Data from both this research and Aarrestad et al. [14] suggest that more modern devices have less drift than those used previously (median (IQR) = −4 (−6 to −2.5) mmHg [15] and 5.1 (1.2 to 7.6) mmHg [9]).

### Comparison of drift measurement: Use of CAPgas versus ABG

Using CAPgas or ABG as the reference value for determining TcCO_2_ drift produced similar results. Both methods revealed a downward drift in TcCO_2_ over the night of a similar mean difference (−0.3 mmHg), despite the absolute offsets differing slightly between the techniques. Individual variability is demonstrated in Fig. 4, with 95% of observations lying within limits of agreement of −6.9 to 6.3 mmHg [2]. We observed no difference in the agreement between CAPgas or ABG sampling over the observed drift range.

### Clinical considerations

Clinical indication, study purpose, funding and care models, clinician and patient preference, staffing expertise, and sleep laboratory capacity may all affect decisions regarding the importance of TcCO_2_ monitoring on any individual patient on any given night. Whether TcCO_2_ is a necessary or desirable inclusion in overnight PSG was not the focus of this paper. Rather we sought to understand the place of arterialised capillary blood gas (CAPgas) sampling to estimate TcCO_2_ drift during laboratory PSG, and its technical success and acceptability in a population with chronic respiratory failure. We have provided our data regarding absolute agreement between ABG, TcCO_2_, and CAPgas values of CO_2_, O_2_ or pH in the Online Tables for information, but this paper does not add new findings with respect to these measurements to the existing, meta-analysed literature [8, 12]. 

## Conclusions

We confirmed recent findings [14] that modern TcCO_2_ devices are more accurate both as a spot measure of CO_2_ and during extended sleep monitoring. As Figures S2a and S2b illustrate however, individual measurement limits of agreement may approach 10mmHg. If a spot blood gas measurement of CO_2_ is desired, a CAPgas may be sufficient, acknowledging a small bias, but if a precise PaO_2_ is clinically necessary, an ABG is indicated. Our data suggests that both CAPgas and ABG measures of CO_2_ are similarly acceptable for estimating overnight TcCO_2_ drift, despite the larger average absolute offset with CAPgas. CAPgas was quicker, less painful and preferred overall by scientists and patients.

## Electronic Supplementary Material

Below is the link to the electronic supplementary material.

Supplementary Material 1 (DOCX 950 KB)

## Data Availability

The data that support the findings of this study are not openly available as participant approval for sharing of raw data was not obtained at time of consent. Reasonable data sharing requests will however be considered by the corresponding author. Any reasonable request for data sharing would require consideration by the local institutional ethics committee. Word count. 3040.
