# Supplementary material for: Capillary versus arterial blood gases. Accuracy, acceptability, and suitability for use during sleep studies to calibrate transcutaneous carbon dioxide measurement
Source: Sleep Breath. 2026 Jul 8;30(4):211. doi: 10.1007/s11325-026-03739-3 (PMC13346311; doi:10.1007/s11325-026-03739-3)
Supplement: Supplementary file 1 — Supplementary Material 1 (DOCX 950 KB) [file 11325_2026_3739_MOESM1_ESM.docx]

# Online Supplement: CAPgas paper

## Authors:

Nicole L Sheers, Linda Rautela, Steven James Lindstrom, Krisha Saravanan, Jennifer Cori, Danny Brazzale, Elisa San Pedro, Michelle Burns, Christine McDonald, Mark E Howard, David J Berlowitz

# Supplemental Results

### Online Supplement Results of measurement agreement between arterial and capillary sampling methods: pO_2_ and pH

For the 57 participants with paired CAPgas and ABG samples at baseline (Evening), the mean difference (95% CI) between arterial and capillary blood gas parameters were: pH -0.019 (-0.023, -0.016), *p*<0.001; PCO_2_ = 2.8 (2.3, 3.3) mmHg, *p*<0.001 and PO_2_ = -2.9 (-4.6, -1.1) mmHg, *p*=0.002. There was a significant correlation between the two sampling methods for pH, CO_2_ and O_2_ values (ICC [95% CI] for pH = 0.90 [0.84, 0.94], p<0.001; CO_2_ = 0.96 [0.94, 0.98], p<0.001; O_2_ = 0.91 [0.85, 0.94], p<0.001). Agreement between ABG and CAPgas pH and O_2_ parameters are illustrated below. Agreement between ABG and CAPgas pCO_2_ is provided in the main manuscript (Figure 2).

|  |  |
| --- | --- |
| **Fig S1a** | 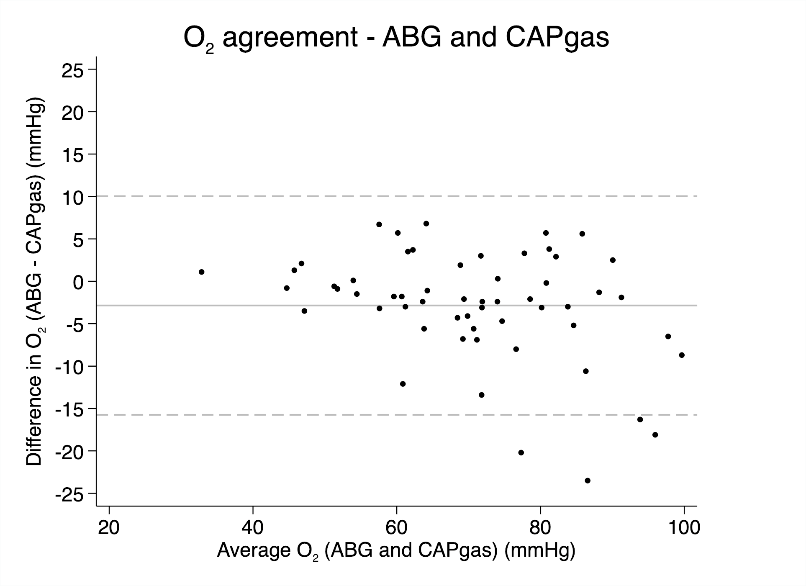 |
|  | Mean bias (limits of agreement) = -2.86 (-15.49 to 9.78) mmHg |
| **Fig S1b** | 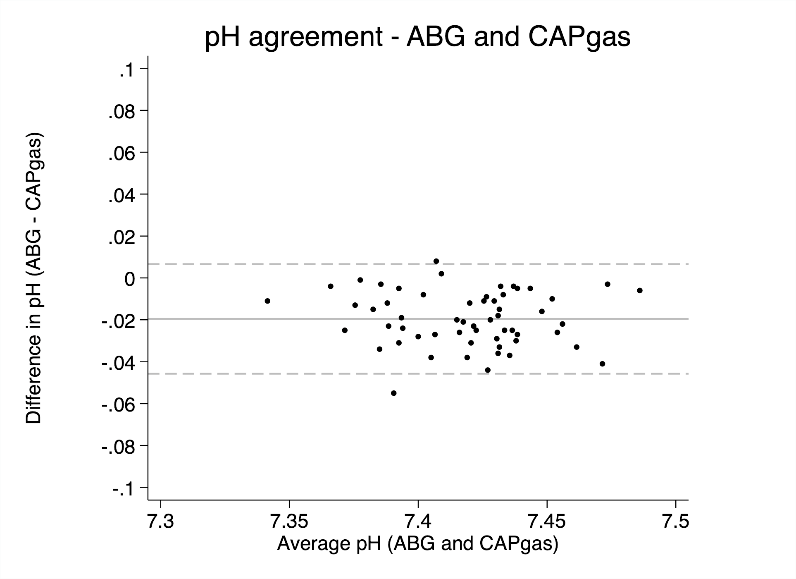 |
|  | Mean bias (limits of agreement) = -0.019 (-0.045 to 0.006) |

**Online Supplement Figure S1: Bland Altman plots illustrating mean difference and limits of agreement between ABG and CAPgas blood parameters for a) O_2_ and b) pH, based on paired samples obtained at the Evening session (n=57).**

*Solid line represents mean bias (mean difference), dashed lines represent upper and lower levels of agreement (mean difference ± 2 standard deviations).*

### Online Supplement Results measurement agreement between blood and transcutaneous sampling methods

Offsets (difference between transcutaneous and arterial or capillary blood sampling technique) were calculated for the 44 participants with all of ABG (PaCO_2_), CAPgas (P_CAP_CO_2_) and TcCO_2_ measures at both Evening and Morning (main manuscript Table 2, Evening data depicted in Online supplement Figures S2a and S2b).

The average absolute PaCO_2_ and P_CAP_CO_2_ values did not change over the night but the TcCO_2_ significantly decreased. There was a larger, negative offset between TcCO_2_ and ABG compared to TcCO_2_ and CAPgas at both Evening and Morning (main manuscript Table 2, Figure 3, Online supplement S2a and S2b, S3). The TcCO_2_ measure drifted further negative from both the ABG and the CAPgas PCO_2_ values over the night, but there was no difference in the magnitude of these drifts between the ABG and the CAPgas sampling methods (main manuscript Table 2, Figure 4).

| **Fig S2a** | 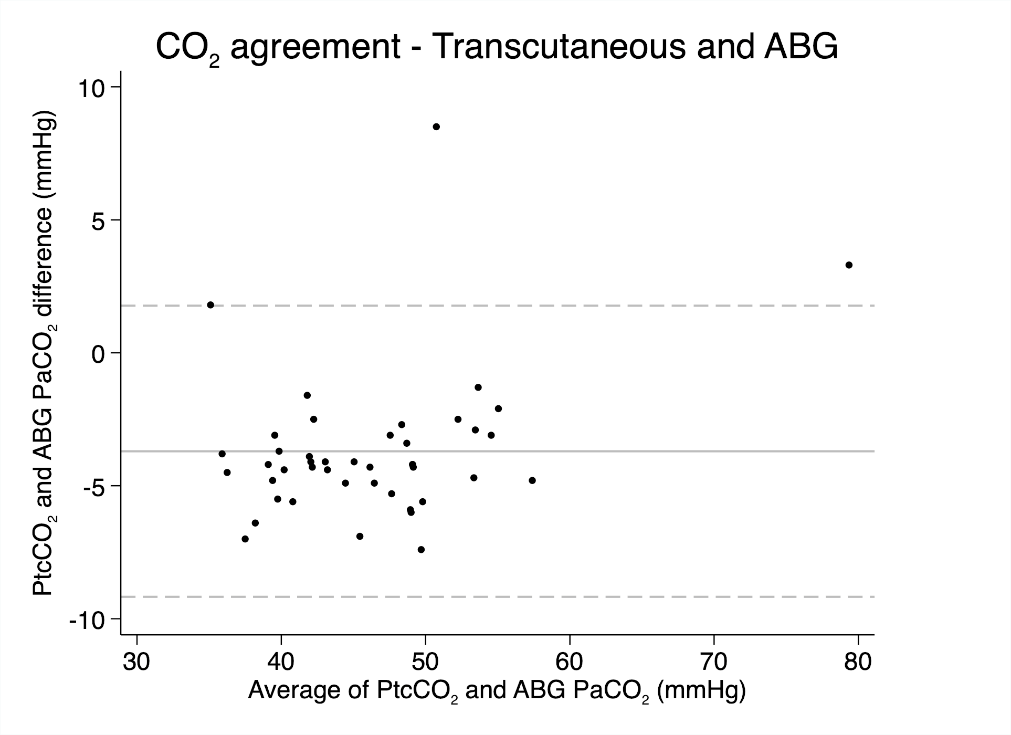 |
| --- | --- |
|  | Mean bias (limits of agreement) = -3.7 (-9.2 to 1.8) mmHg |
| **Fig S2b** | 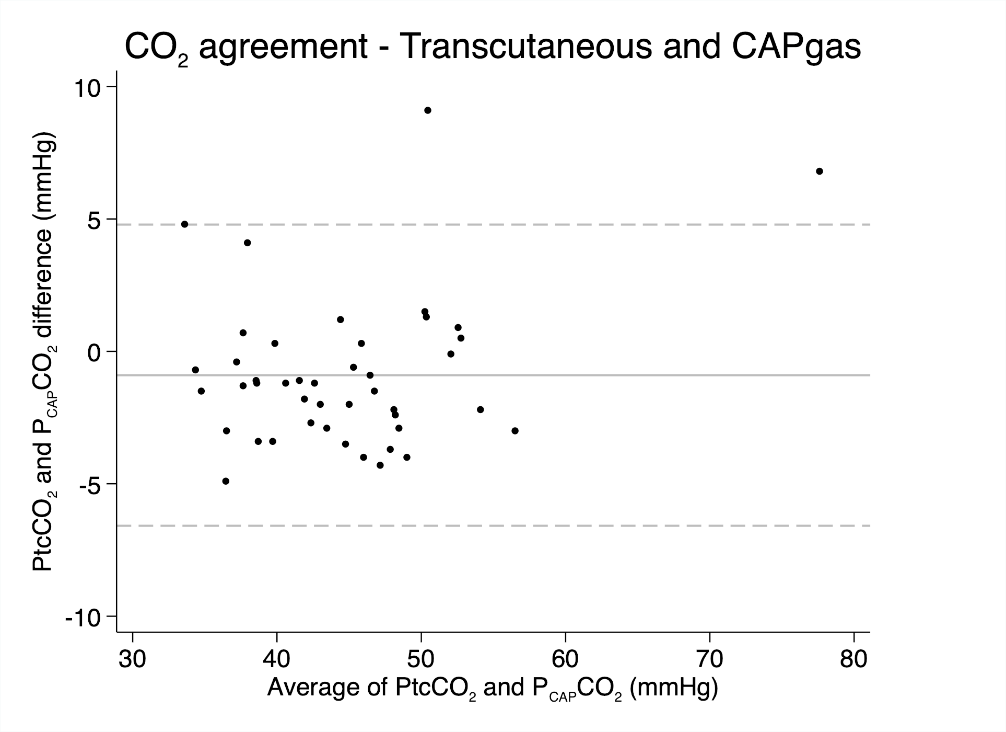 |
|  | Mean bias (limits of agreement) = -0.9 (-6.6 to 4.8) mmHg |

**Online Supplement Figure S2: Bland Altman plots illustrating Offset: mean difference and limits of agreement between a) transcutaneous (PtcCO_2_) and arterial (ABG PaCO_2_) or b) transcutaneous (PtcCO_2_) and capillary (CAPgas P_CAP_CO_2_) blood gas values.**

**Data are individual participant paired samples obtained at the Evening session (n=44)**

*Solid line represents mean bias (mean difference: transcutaneous minus ABG or CAPgas), dashed lines represent upper and lower levels of agreement (mean difference* ± *2 standard deviations).*

| **Fig S3** | 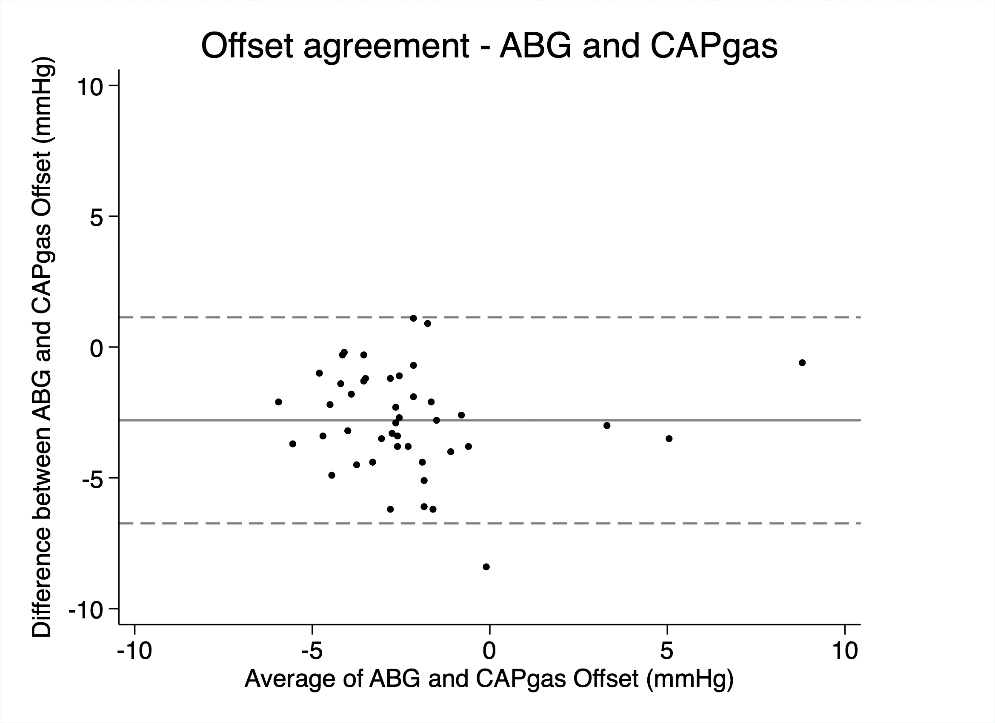 |
| --- | --- |

**Online Supplement Figure S3: Bland Altman plot illustrating mean difference and limits of agreement between the ABG Offset (difference between transcutaneous (PtcCO_2_) and arterial (ABG PaCO_2_)) and the CAPgas Offset (difference between transcutaneous (PtcCO_2_) and capillary (CAPgas P_CAP_CO_2_)). Data are individual participant paired samples obtained at the Evening session (n=44).**

*Solid line represents mean bias (mean difference: ABG offset minus CAPgas offset), dashed lines represent upper and lower levels of agreement (mean difference ± 2 standard deviations = -2.80 ± 3.94 [-6.74, 1.14]).*

| **Fig S4a** | 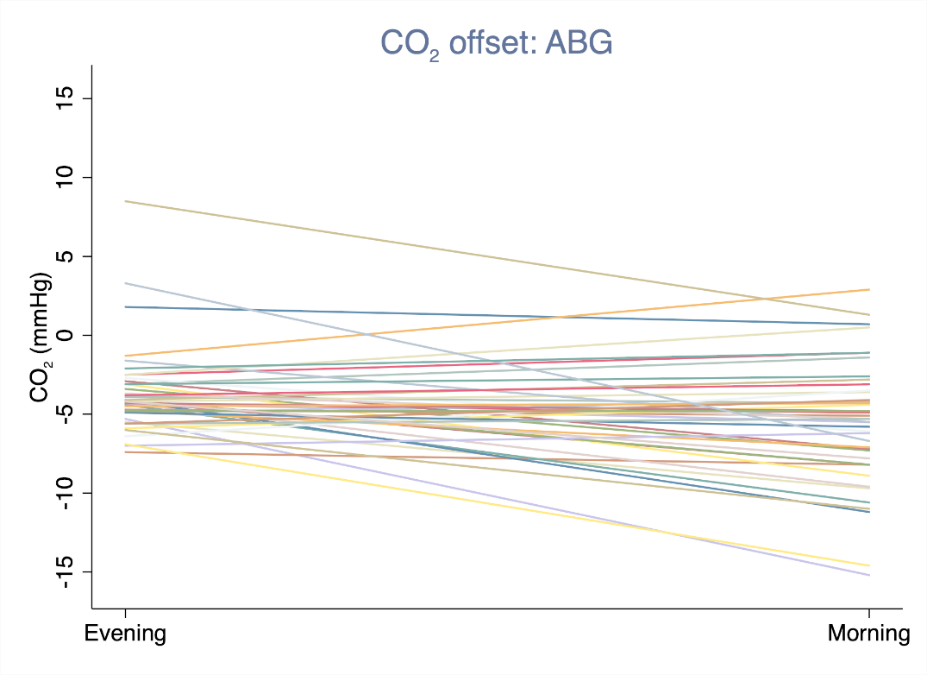 |
| --- | --- |
|  |  |
| **Fig S4b** | 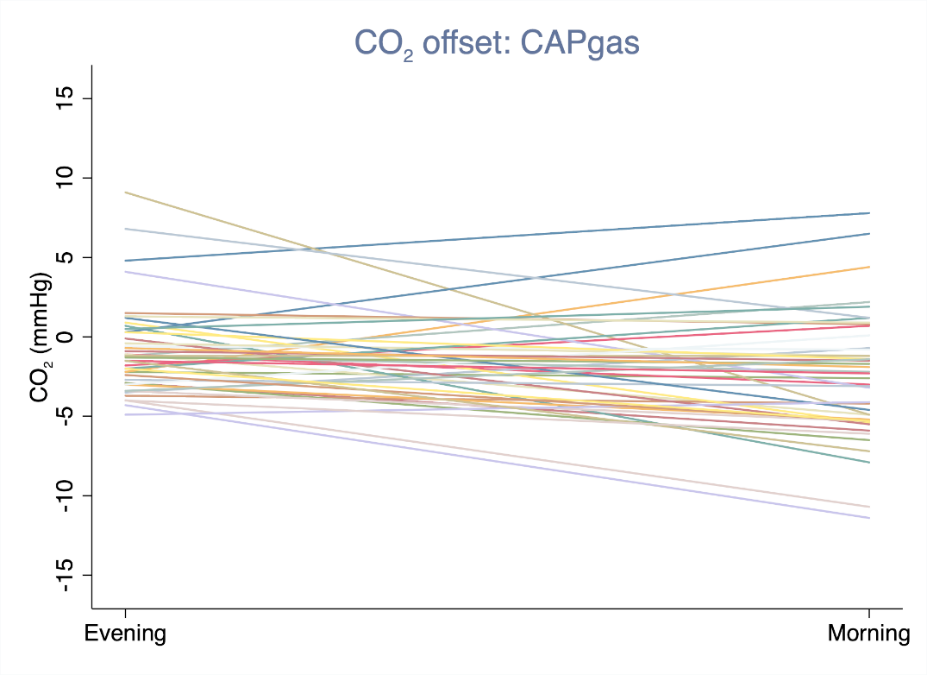 |

**Online Supplement Figure S4: Individual participant data illustrating ABG Offset (Fig S4a) and CAPgas Offset (Fig S4b) at Evening and Morning (n=44)**

*Where offset is the difference between transcutaneous and blood sampled carbon dioxide, E.g.: ABG Offset = PtcCO_2_ _ABG_ - PaCO_2_; CAPgas Offset = PtcCO_2 CAP_ - P_CAP_CO_2_.*

PtcCO_2 ABG_ *= transcutaneous carbon dioxide at time of ABG;* PaCO_2_ *= carbon dioxide on arterial blood gas (ABG);* TcCO_2 CAP_ *= transcutaneous carbon dioxide at time of CAPgas;* P_CAP_CO_2_ *= carbon dioxide on capillary blood gas (CAPgas).*

### Online Supplement Results of participant and scientist preferences

Backward stepwise logistic regression models to investigate the influence of number of CAPgas attempts, number of ABG attempts, CAPgas sampling duration, ABG sampling duration, pain with CAPgas, pain with ABG, and scientist experience on i) Scientist preference, and ii) Participant preference were performed (where CAPgas is preferred method).

| Scientist Preference | Odds-ratio | Lower 95% CI | Upper 95% CI | p-value |
| --- | --- | --- | --- | --- |
| Scientist experience | 1.07 | 1.02 | 1.13 | 0.012 |
| CAPgas sampling duration | 0.99 | 0.98 | 1.00 | 0.108 |
| CAPgas pain | 0.81 | 0.62 | 1.07 | 0.137 |
| Constant | 1.81 | 0.22 | 14.9 | 0.581 |

Overall model χ^2^ = 21.72, p<0.001

| Participant Preference | Odds-ratio | Lower 95% CI | Upper 95% CI | p-value |
| --- | --- | --- | --- | --- |
| ABG pain | 2.22 | 1.43 | 3.48 | <0.001 |
| CAPgas pain | 0.52 | 0.36 | 0.76 | 0.001 |
| Constant | 1.22 | 0.47 | 3.17 | 0.681 |

Overall model χ^2^ = 28.44, p<0.001

**Online Supplement Table S1: Logistic regression models of Scientist and Participant Preferences for sampling technique.**
